# Adult-type rhabdomyoma of the thyroid: A case report

**DOI:** 10.3389/fonc.2023.1108133

**Published:** 2023-01-18

**Authors:** ZhenPeng Jiang, MengNi Zhang, JiaYan Huang, Ling Song, Qiang Lu

**Affiliations:** ^1^ Department of Medical Ultrasound, West China Hospital of Sichuan University, Chengdu, Sichuan, China; ^2^ Department of Pathology, West China Hospital of Sichuan University, Chengdu, Sichuan, China

**Keywords:** rhabdomyoma, adult-type, thyroid, ultrasound, case

## Abstract

Adult-type rhabdomyoma (AR) is a benign myogenous neoplasm. It is rarely located in the thyroid. We present a case of a 61-year-old man, presenting with complaints of a mass found in his left neck for three years. Ultrasonography and computed tomography showed a mass in the left lobe of the thyroid. Subsequently, a fine-needle aspiration biopsy showed that the lesion was suspected to be an oncocytic neoplasm, and the patient underwent surgery. Finally, the lesion was confirmed to be an AR of the thyroid by postoperative pathological diagnosis. In conclusion, AR that occurs in the thyroid is remarkably rare. No case reports to date have described in detail the imaging findings of AR in the thyroid. This study demonstrates the imaging characteristics of a patient with AR of the thyroid, in order to provide more extensive insights to consider the differential diagnosis of thyroid lesions.

## 1 Introduction

Adult-type rhabdomyoma (AR) is an extremely rare benign tumor accounting for less than 2% of myogenous neoplasms (1, 2). The majority of ARs (about 90%) occur in the head and neck (3). Due to extremely low incidence, lack of unique clinical manifestations and typical imaging features, the diagnosis of AR mainly relies on characteristic histopathologic and immunohistochemical features (4). There are a few studies reporting that AR can be misdiagnosed as thyroid nodule, but AR arising from the thyroid is extremely rare (5–7). To our knowledge, it is the first case of AR originating from the thyroid that was reported in English literature.

## 2 Case report

**Figure 1:**
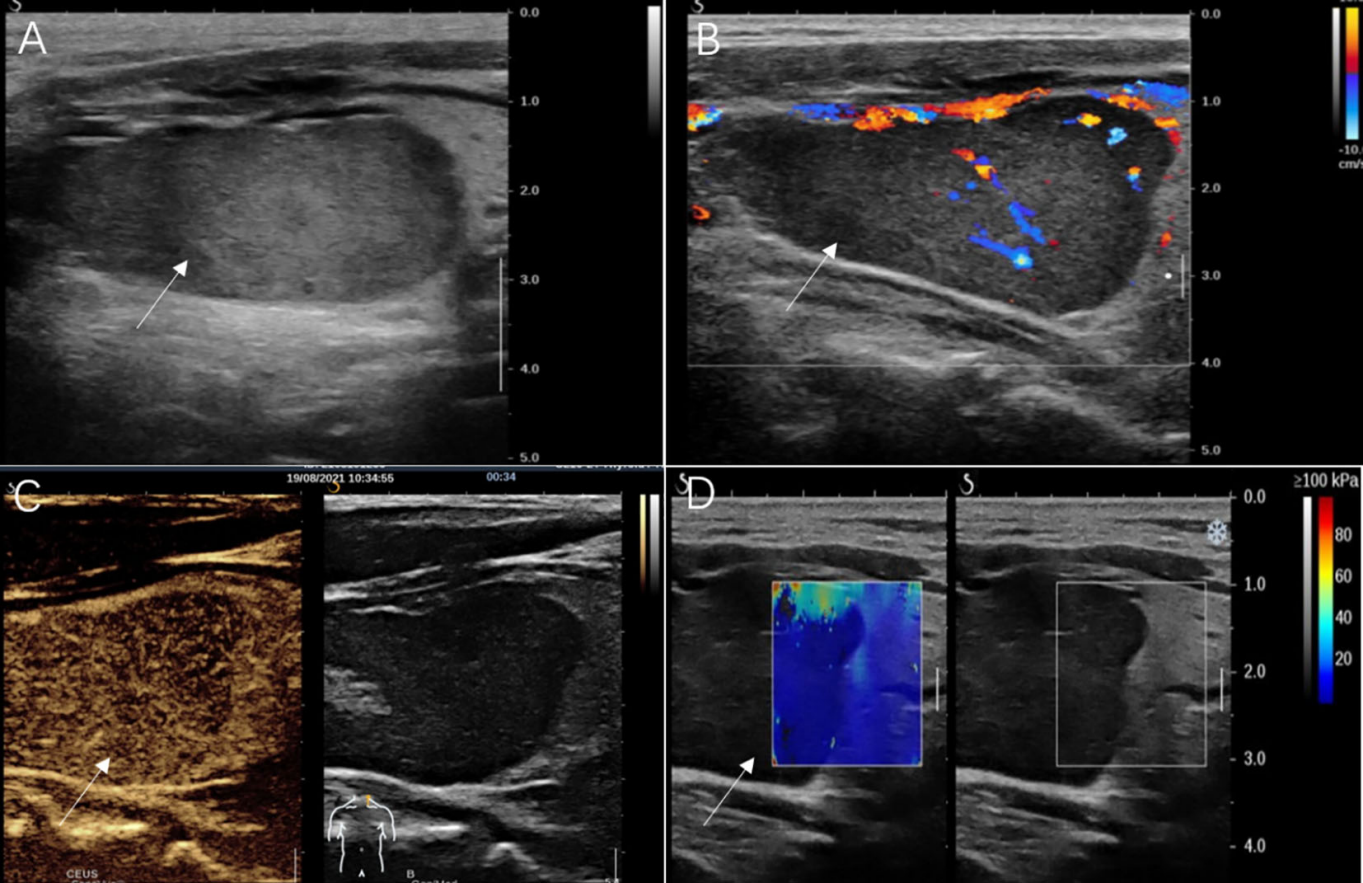
Ultrasonography images of adult-type rhabdomyoma of the thyroid. **(A)** Conventional gray-scale sonography revealed a solid and hypoechoic thyroid nodule (arrow) in the left lobe. **(B)** Color Doppler flow imaging showed a relatively rich blood flow signal inside this nodule (arrow). **(C)** Contrast-enhanced ultrasound image showed hypoenhancement of the nodule (arrow) in the arterial phase. **(D)** Shear wave elastography showed a lower stiffness of the nodule (arrow) compared with that of the thyroid.

**Figure 2:**
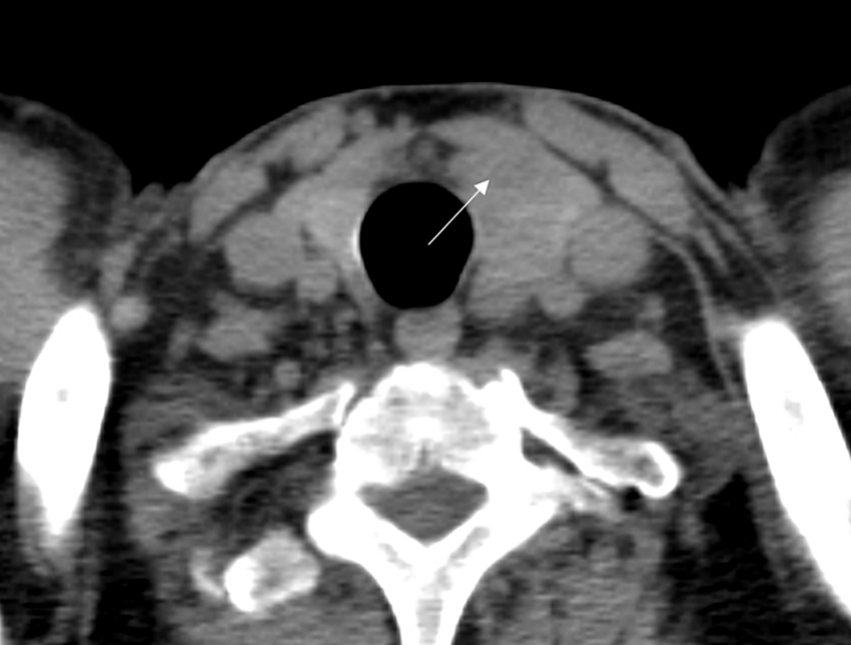
CT images of adult-type rhabdomyoma of the thyroid. Plain CT images revealed the left thyroid lobe was obviously enlarged, and there was a lesion (arrow) with slightly low density in the left thyroid lobe.

A 61-year-old male was referred to our hospital due to a mass found in his left neck three years ago. The patient complained no salient clinical symptoms, including tachycardia, sweating, weight loss, dyspnea, or hoarseness. He had no history of neck radiation or familial thyroid disease. A moderately hard and painless nodule measured 5 cm in the left thyroid lobe that could move with swallowing was detected by physical examination. Meanwhile, routine blood tests, thyroid hormone, and thyroid-stimulating hormone were within the normal ranges. A hypoechoic solid nodule (measured 50 mm× 23 mm × 23 mm) with a clear boundary was found in the enlarged left lobe of the thyroid, and punctate echogenic foci was not detected on ultrasonography (**Figure 1A**). According to ACR TI-RADS (American College of Radiology, Thyroid Imaging Reporting and Data System), this nodule was assigned to TR-4 category. Blood flow signals within and around the nodule were shown by Color Doppler flow imaging (**Figure 1B**). The patient underwent follow-up instead of surgical treatment. However, the gradual growth during the follow-up was presented by semiannual thyroid ultrasonography. Therefore, further evaluation was recommended. A fine-needle aspiration (FNA) biopsy under the guidance of ultrasound was performed, and the lesion was suspected to be an oncocytic neoplasm by pathology. Furthermore, the contrast-enhanced ultrasound (CEUS) was performed with a bolus injection of 2 mL of SonoVue (Bracco, Milan, Italy) followed by 5 mL of saline. The lesion showed heterogeneous hypoenhancement during the whole procedure of CEUS (**Figure 1C**). Moreover, the lesion was soft according to the stiffness measurement by shear wave elastography (SWE) (Emax=5.3 kPa, Emean=4.2 kPa, Emin=3.2 kPa, Ratio=0.4) (**Figure 1D**). The lesion showed slightly low density with unclear boundary in the enlarged left thyroid lobe on plain computed tomography (CT) (**Figure 2**).

**Figure 3:**
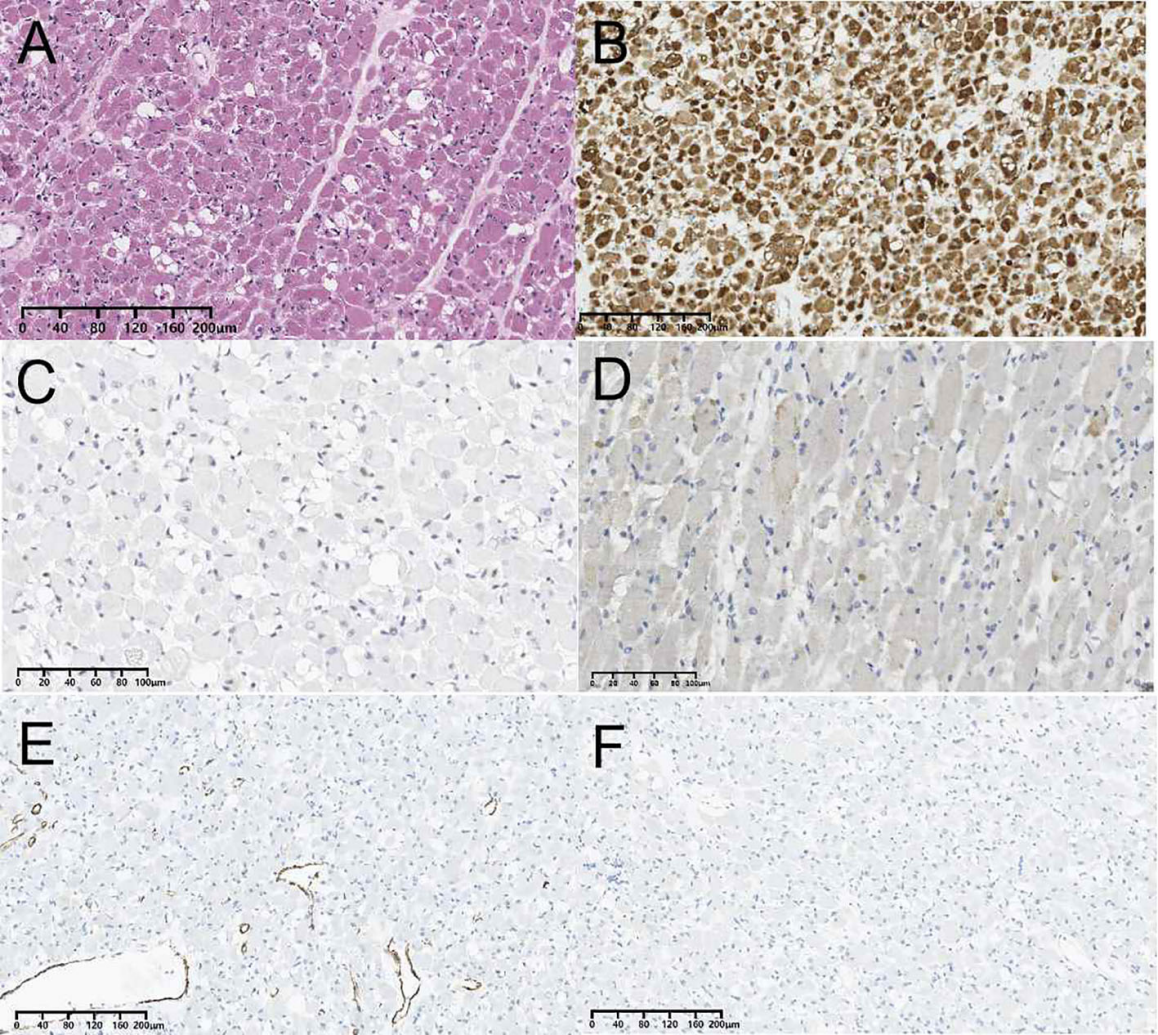
Pathological findings of adult-type rhabdomyoma of the thyroid. **(A)** Histopathological section (hematoxylin and eosin staining). **(B)** Immunohistochemistry result showing the expression of desmin. **(C)** Immunohistochemistry result showing the expression of MyoD1. **(D)** Immunohistochemistry result showing the expression of myogenin. **(E)** Immunohistochemistry result showing the expression of smooth muscle actin. **(F)** Immunohistochemistry result showing the expression of S-100 protein.

Ultimately, the patient underwent resection of the left thyroid lobe and isthmus due to the suspicious malignant nodule. The patient exhibited normal preoperative thyroid hormone, thyroglobulin, and thyroid-stimulating hormone level. During surgery, the trachea was found to be pushed to the unaffected side of the neck due to the enlarged left lobe and isthmus of the thyroid. The tumor of the left thyroid lobe was a red, soft, and solid mass with a clear boundary. The central neck lymph node dissection was performed, and no enlarged lymph nodes were found visually during surgery. Postoperative pathology showed lymph node metastasis was not found. Histologic examination of the tumor showed that it consisted of bland spindle cells, immature elongated cells with bipolar cytoplasmic extensions, strap-type rhabdomyoblasts with abundant eosinophilic cytoplasm and round vesicular nuclei displaying a fascicular growth pattern (**Figure 3A**). No pleomorphism, necrosis, or atypical mitoses were observed. On immunohistochemistry, the tumor had strong desmin (**Figure 3B**) and weak sparse myoD1 expression (**Figure 3C**), while myogenin (**Figure 3D**), smooth muscle actin (**Figure 3E**), S-100 protein (**Figure 3F**), thyroglobulin, thyroid transcription factor 1, cytokeratin, and paired box protein 8 were negative. The expression of Ki-67 was less than 1%. Finally, the lesion was confirmed to be an AR of the thyroid by histopathology. After an uneventful postoperative course, the patient was discharged. No postoperative complications, such as hoarseness and hypoparathyroidism, were reported after the surgery. The patient is currently well one year after surgery and is undergoing semiannual conventional ultrasound examinations to follow up.

## 3 Discussion

AR is a rare benign tumor which has a much lower incidence than its malignant counterpart, rhabdomyosarcoma (1). Most of ARs are solitary (70%) and generally afflict males over 50 years old (8). The head and neck are the most frequently involved sites, followed by the extremities, esophagus, stomach, mediastinum, orbit, prostate, and intracranial area (8). To our knowledge, this is the first reported case of an AR in the thyroid, although AR occurring around the thyroid was previously reported (5–7). Previous studies have reported that rhabdoid cells could be detected in various types of thyroid malignancies dominated by undifferentiated thyroid carcinoma (9, 10). The presence of rhabdoid cells has been considered to be a poor prognostic factor (11). Nonetheless, the tumor entity of rhabdoid cells in the thyroid has not been previously reported. Additionally, primary thyroid rhabdomyosarcoma has been reported (12). Willrich et al. (13) reported a case of AR at the submandibular gland. However, we report the first benign case of a rhabdomyoma arising from the thyroid.

Although AR is a benign entity, 42% of ARs may still recur owing to incomplete resection (14). Moreover, residual tumors may have a malignant potential (15). Therefore, accurate preoperative diagnosis of AR is crucial. However, due to the extremely low incidence of AR in the thyroid, AR may be misdiagnosed as other common types of thyroid entities by pathologists without significant experience (5). In our case, the patient underwent FNA biopsy, and the lesion was suspected to be an oncocytic neoplasm by pathology. Meanwhile, abundant eosinophilic cytoplasm in the tumor cells of the AR has also been reported by previous literature (8). It is possible that AR and oncocytic neoplasm may show similar FNA biopsy results (16). Compared with AR, oncocytic neoplasm occurs more commonly in the thyroid (17). Therefore, it is difficult for pathologists to diagnose an AR originating from the thyroid by FNA biopsy.

Current diagnostic imaging modalities, such as ultrasound, CT, and MRI, have not been demonstrated to be highly specific for the diagnosis of AR (1). The lesion showed a wider-than-tall shape, distinct margin, and homogeneous internal echo on conventional ultrasound images. These features were also consistent with ARs detected in other sites according to previous reports (5, 18). When evaluated by SWE, the lesion was softer than the surrounding normal thyroid tissue. Moreover, although the lesion was large, cervical lymphadenopathy, contour bulging of the mass, and loss of the echogenic thyroid border were not detected. The above sonographic appearances of the lesion were similar to those of benign lesions. However, a solid hypoechoic nodule, abundant blood flow signals, and heterogeneous hypoenhancement on CEUS are still prone to a malignant lesion. The CEUS findings of AR have not yet been reported. In this case, the lesion showed hypoenhancement on CEUS images. It may be explained by the fact that the thyroid gland is an organ with abundant blood supply (19). In this case, plain CT indicated that the left thyroid lobe was obviously enlarged, and a lesion with slightly low density was detected in the left thyroid lobe (mean CT value of the lesion, 64HU; mean CT value of surrounding normal tissue, 81 HU). The lesion had an unclear boundary which is in line with AR occurring in extra thyroid sites according to previous literature (20, 21). Unfortunately, contrast-enhanced CT and MRI were not performed in our case.

Differential diagnoses should be made with other primary tumors of the thyroid, including thyroid follicular neoplasms (FNs), thyroid Hürthle cell neoplasms (HCNs), and primary thyroid lymphomas (PTLs). **Table 1** summarizes the sonographic appearances of AR and other thyroid tumors. FNs of the thyroid gland include follicular thyroid adenoma (FTA) and follicular thyroid carcinoma (FTC). However, based on conventional ultrasound or CEUS, it is difficult to distinguish FTC from FTA (19, 22). This case showed a well-defined, solid, and hypoechoic nodule on conventional ultrasound, which was similar to that of FNs (23, 24). Meanwhile, this case appeared as a soft lesion on SWE, which was also a feature similar to FNs (19). However, prior small sample studies reported that FNs could show homogeneous hyperenhancement or a regular high-enhancing ring on CEUS (22, 25, 26). The enhancement pattern was different from our case. Therefore, thyroid CEUS may be helpful for the differential diagnosis. HCNs (also called oncocytic cell tumors) are rare tumors characterized by the presence of more than 75% oncocytic cells (27). HCNs used to be classified as a variant of FNs (24). However, HCNs and FNs are recognized as two separate entities in the latest WHO classification (17, 28). HCNs may have a variety of sonographic appearances. However, a mixed echo nodule with medium and low echoes is the most common ultrasonogram performance of HCNs (29). Lee et al. (30) reported the incidence of cystic component was 44.4% in HCNs. The cystic areas could be gradually replaced by hypoechoic solid contents, and the lesion finally formed a completely solid echo nodule (29). Furthermore, HCNs are deemed to be soft on SWE (19). However, the CEUS appearance of HCNs has not been previously reported. Therefore, it is challenging to distinguish HCNs from our case by ultrasound. The most common symptom of PTLs is a rapidly growing painless goiter that can be accompanied by dyspnea, dysphagia, and hoarseness (31). The majority of these cases are patients with Hashimoto’s thyroiditis. In conventional ultrasound, the appearances such as marked hypoechogenicity, posterior acoustic enhancement, and hypervascularity may suggest a PTL (32). In addition, most PTLs show heterogeneous hypo‐enhancement on CEUS (33). As our case showed similar appearances on CEUS as that of the PTL. Therefore, conventional ultrasound and clinical presentation may be more useful for making a differential diagnosis between the two entities.

**Table 1:** The sonographic appearances of AR and other thyroid tumors.

Entities	Details
AR	A well-defined, hypoechoic, and solid nodule appears as a soft lesion on SWE and shows heterogeneous hypoenhancement on CEUS.
FN	A hypoechoic solid nodule with clear boundaries is soft on SWE and shows homogeneous hyperenhancement or a regular high-enhancing ring on CEUS.
HCN	A mixed echo lesion with medium and low echoes is the most common and the lesion is deemed to be soft on SWE.
PTL	The lesion with marked hypoechogenicity, posterior acoustic enhancement, and hypervascularity mostly occurs in the patient with Hashimoto’s thyroiditis and shows heterogeneous hypoenhancement on CEUS.

AR, Adult-type rhabdomyoma; FN, Follicular neoplasm; HCN, Hürthle cell neoplasm; PTL, Primary thyroid lymphoma; SWE, Shear wave elastography; CEUS, Contrast-enhanced ultrasound.

## 4 Conclusion

AR originating from the thyroid is extremely rare which can also be a pitfall for those common types of thyroid tumors. Multimodal ultrasound is helpful for the diagnosis of AR, and a surgical resection may not be avoided when AR is suspected.

## Data availability statement

The original contributions presented in the study are included in the article/supplementary material. Further inquiries can be directed to the corresponding author.

## Ethics statement

Ethical review and approval were not required for the study on human participants in accordance with the local legislation and institutional requirements. The patients/participants provided their written informed consent to participate in this study. Written informed consent was obtained from the individual(s) for the publication of any potentially identifiable images or data included in this article.

## Author contributions

ZJ prepared the manuscript. MZ was responsible for histology and immunohistochemical images. JH and LS supported the data acquisition and manuscript revision. QL supervised the writing and revision of the manuscript. All authors contributed to the article and approved the submitted version.
